# PREPL Deficiency: A Homozygous Splice Site PREPL Mutation in a Patient With Congenital Myasthenic Syndrome and Absence of Ovaries and Hypoplasia of Uterus

**DOI:** 10.3389/fgene.2020.00198

**Published:** 2020-03-11

**Authors:** Qi Yang, Rong Hua, Jiale Qian, Shang Yi, Fei Shen, Qiang Zhang, Mengting Li, Sheng Yi, Jingsi Luo, Xin Fan

**Affiliations:** ^1^Genetic and Metabolic Central Laboratory, Guangxi Maternal and Child Health Hospital, Nanning, China; ^2^The Guangxi Zhang Autonomous Region Reproductive Hospital, Nanning, China

**Keywords:** PREPL mutation, PREPL deficiency, absence of ovaries and hypoplasia of uterus, congenital myasthenic syndrome, PREPL gene

## Abstract

Prolyl endopeptidase-like (PREPL) deficiency (MIM 616224) is a very rare congenital disorder characterized by neonatal hypotonia and feeding difficulties, ptosis, neuromuscular symptoms, cognitive impairments, growth hormone deficiency, short stature, and hypergonadotropic hypogonadism. This syndrome is an autosomal recessive disease resulting from mutations in the *PREPL* gene. Previous reports have associated PREPL deficiency with only one nucleotide substitution, the deletion of four nucleotides, and eight small microdeletions in the *PREPL* gene In this study, we used whole exome sequencing (WES) to identify a novel homozygous splicing mutation (c.616 + 1G > T) in a 14-year-old Chinese girl with PREPL deficiency. Sequencing of the RT-PCR products from the patient’s blood sample revealed that the c.616 + 1G > T variant disrupted normal splicing in intron 4 leading to an aberrant inclusion of 43 nucleotides in intron, a frameshift, and premature termination codon. Our patient exhibited several of the common phenotypes, including severe neonatal hypotonia, growth impairment and cognitive problems. However, we also observed several unusual phenotypic characteristics: absence of the ovaries, hypoplasia of the uterus, microcephaly and a short neck in patient is alsoobserved. These results provide further evidence for the involvement of PREPL development of the ovaries and uterus. Our findings may provide further insight into the relationship between the genotype and phenotype in collagen-associated diseases and improve the clinical diagnosis of Prolyl endopeptidase-like deficiency.

## Introduction

Hypotonia-cystinuria syndrome (HCS) is a very rare autosomal recessive disorder characterized by cystinuria, severe neonatal hypotonia, growth impairment, and cognitive problems (Jaeken et al., 2006; Martens et al., 2007; Bartholdi et al., 2014). This syndrome is caused by recessive deletions of *SLC3A1* and *PREPL* genes on chromosome 2p21 (Régal et al., 2012). In HCS, *SLC3A1* deficiency, result in cystinuria (MIM 606407) (Font-Llitjós et al., 2005), while *PREPL* deficiency causes other symptoms (Jaeken et al., 2006; Régal et al., 2018). Congenital myasthenic syndrome (MIM 616224) is an autosomal recessive disorder caused by isolated PREPL deficiency. Subjects with isolated PREPL deficiency often experience severe hypotonia during the in neonatal period although this improves spontaneously. In addition, patients can experience muscular weakness, feeding problems, ptosis, growth hormone deficiency, failure to thrive, short stature, and hypergonadotropic hypogonadism (Régal et al., 2014; Silva et al., 2018).

The prolyl endopeptidase like gene, *PREPL* (PREPL; MIM 609557, NM_006036.4) is ∼43 kb long and located in 2p21, and encodes PREPL protein which is a cytoplasmic serine hydrolase structurally belonging to an oligopeptidase family (Szeltner et al., 2005). PREPL plays an important role in regulating the exocytosis of synaptic vesiscles (Engel et al., 2015). To date, only thirteen mutations have been described in the *PREPL* gene that are associated with HCS or congenital myasthenic syndrome (Laugwitz et al., 2018; Silva et al., 2018^1^). Here we report a Chinese female with isolated *PREPL* deficiency, who carried a novel splicing mutation (c.616 + 1G > T) in the PREPL gene. Furthermore, our patient represents the first case of isolated *PREPL* deficiency to exhibit an absence of the uterus and ovaries, microcephaly and a short neck.

## Materials and Methods

### Patients

**FIGURE 1:**
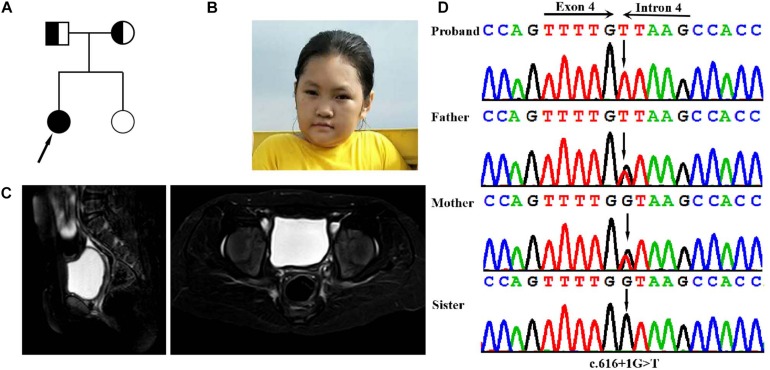
Clinical and genetic features. **(A)** Pedigree chart of the family of the isolated PREPL deficiency patient. **(B)** Facial photographs of the patient at the age of 14 years: showing microcephaly, facial weakness, mild attenuation of palpebral ptosis and micrognathia, and short neck. **(C)** T2WI sagittal and axial sections showing absent uterus and hypoplasia of uterus in the pelvis the. **(D)** DNA sequence chromatograms by Sanger sequencing of PREPL showing a homozygous splicing mutation c.616 + 1G > A in the proband. Sanger sequencing further revealed that her parents were heterozygous for the same mutation, and that her sister was normal.

We recruited a family featuring isolated PREPL deficiency family (Figure 1A). All of the participants undergo genetic analysis and provided informed written consent. The study was approved by the Department of Genetic Metabolic Central Laboratory of Guangxi Zhuang Autonomous Region, Women and Children Care Hospital.

## Genetic Analysis

### Whole-Exome Sequencing and Sanger Sequencing

Whole exome sequencing (WES) was performed using the genomic DNA extracted from proband (Figure 1B) with an Agilent SureSelect Human All Exon V5 Kit (Agilent Technologies, Santa Clara, CA, United States) for target capture. The library was sequenced on a Hiseq2500 platform (Illumina, San Diego, CA, United States) in accordance with the manufacturer’s instructions. The sequencing reads were aligned to GRCh37.p10 using Burrows-Wheeler Aligner (BWA) software. A custom pipeline mainly built on GATK was used for sequence data analysis and annotation (McKenna et al., 2010). Identification of the causal variant was facilitated by TGex software (LifeMap Sciences, United States), which was used to annotate the selected SNVs and indels. “Rare deleterious” mutations were defined as those that met the following criteria: (a) they led to a stop-gain, stop-loss, non-synonymous, frameshift or splice-site mutation; and (b) their alternative allele frequencies were each equal to or <0.5% in the gnomAD database.

### *In silico* Analysis

SIFT,^2^ PolyPhen 2.0^3^ and Mutation Taster^4^ were performed in order to calculate the pathogenicity index of all novel missense variants with unknown clinical significance. Splice sites were prediction by the Human Splicing Finder website.^5^ The candidate *PREPL* variant was validated by Sanger sequencing and its pathogenicity was classified following to ACMG/AMP guidelines (Richards et al., 2015).

### RNA Analysis

Abnormal PREPL splicing of PREPL mRNA was identified and analyzed by RT-PCR using RNA obtained from the venous blood of the patient, her parents and normal controls according to the instructions of a QIAamp RNA Blood Mini kit (Qiagen GmbH, Hilden, Germany). cDNA synthesis was carried out using the PrimeScript^TM^ RT reagent Kit (Takara Bio Inc). RT-PCR amplification was then carried out to identify normal or abnormal PREPL splicing using specific primers that spanned the region from exon 3 to exon 5: 5′-AGGTTGTTGCTTGGTTCGTT-3′ (sense) and 5′-AGG ACCCCATGTATTCGCTT-3′ (antisense). The resultant amplicons were then verified by Sanger sequencing.

## Results

### Patient Description

The proband was born at full term with a normal birth weight. She was born as the first child of healthy, non-consanguineous Chinese parents. She was admitted to hospital due to feeding problems, mild respiratory distress, and stridor, when she was 3-days-old. She began walking at the age of 30 months, and exhibited hypotonia but improved afterward. Unfortunately, although the feeding difficulties and hypotonia had continued, these problems were not taken seriously and the patient no longer received systematic examination and treatment. However, in November 2018, at 14 years-of-age, the proband was admitted to the Guangxi Zhuang Autonomous Region Women and Children Care Hospital for genetic counseling due to intellectual disability (ID), short stature, and primary amenorrhea. Physical examination showed that she presented with severe short stature (height: 125 cm,-6SD), her BMI index was normal (weight: 29.1 kg (<2SD), BMI: 18.56 kg/m^2)^. Her facial dysmorphic features included microcephaly (50 cm, <2SD), facial weakness, mild attenuation of palpebral ptosis and micrognathia, and other deformities including a short neck (Table 1). Physical examination showed that the patient’s breasts were at Tanner stage 1: she had no armpit hair or pubic hair, although her external genital formation was normal. Magnetic resonance imaging (MRI) of the abdomen confirmed the absence of the ovaries and hypoplasia of the uterus (Figure 1C). Motor examination revealed that, she had global muscle weakness and hyporeflexia. She was was assessed on the Chinese Wechsler Intelligence Scale for Children at the age of 14. Her Full Scale IQ was 80 and her verbal comprehension index was 75; her processing speed index was 60. Her communication was poor and she was particularly weak in terms of numbering, maths, English, and visual spatial capability. A growth hormone (GH) provocative test using arginine and L-dopa revealed a peak GH value of 2.19 ng/ml (0.30 ng/ml at 0 min, 0.09 ng/ml at 30 min, 0.26 ng/ml at 60 min, 0.52 ng/ml at 90 min, and 2.19 ng/ml at 120 min), thus suggesting a partial growth hormone deficiency. Levels of follicle stimulating hormone (16.37 IU/L), and estradiol (54 pmol/L), further suggested that the patient had hypergonadotropic hypogonadism. Her karyotype was determined to be a female (46, XX).

**TABLE 1:** Clinical features of the patient with a novel PREPL mutation in homozigosity.

**Clinical features**	**Patient**
Variants in PREPL (NM_006036.4)	c.616 + 1G > T
Gender	Female
Age at last examination	14 year
Gestation	Full-term
Birth weight	2.5 kg (<-2SD)
Birth length	45 cm (<-2SD)
Feeding difficulty	Yes
Muscular hypotonia	Yes
Weight	29.1 kg (<-2SD)
Height	125 cm (<-6SD)
BMI (kg/m2)	18.56
OFC	50 cm (<-2SD)
Developmental delay/intellectual disability/movement delay	Yes; cognitive impairment
Age of walking	30 months
Age of first words	15 months
Behavior anomalies	Poor eye contact; Sensitive;
Brain anomalies	MRI normal
Abnormal eyelid morphology	mild attenuation of palpebral ptosis
Hearing	Normal
Urogenital anomalies	absence of ovaries and hypoplasia of the uterus
Stages of mammary development	B1
Pubic hair	no pubic hair
Facial dysmorphisms	microcephaly, facial weakness, micrognathia flat malar bone; micrognathia
Other anomalies	Short neck

### Mutation Analysis

Genomic DNA was extracted from peripheral blood samples of the proband. A total of >99% of reads were mapped to genomic targets, with 20× coverage for >97% of bases. In the proband, a total of 15340 variants were identified in exonic and splice site regions. We identified 1132 variants with a minor allele frequency (MAF) in gnomAD database that was <0.05. We then used ClinVar to omit all neutral and benign variants. Using TGex software (LifeMap Sciences, United States), we identified 8 variants existed in genes that matched with known phenotypes. Variants from 6 genes associated with *PREPL, GLI2, PTCH1, JAG1, COG4*, and *ARID1A* were subsequently extracted, leading to the identification of a novel homozygous splicing mutation (c.616 + 1G > T) in the *PREPL* gene (RefSeq NM_006036.4) that co-segregated with the disease phenotype observed in our study family (Figure 1D). Sanger sequencing further revealed that her parents were heterozygous for the same mutation, and that her sister was normal (Figure 1D). The variant (rs1165807015) is listed in gnomAD (1 mutated allele out of 31380, seen in genome samples where it failed the random forest filters and not detected in exome samples.

**FIGURE 2:**
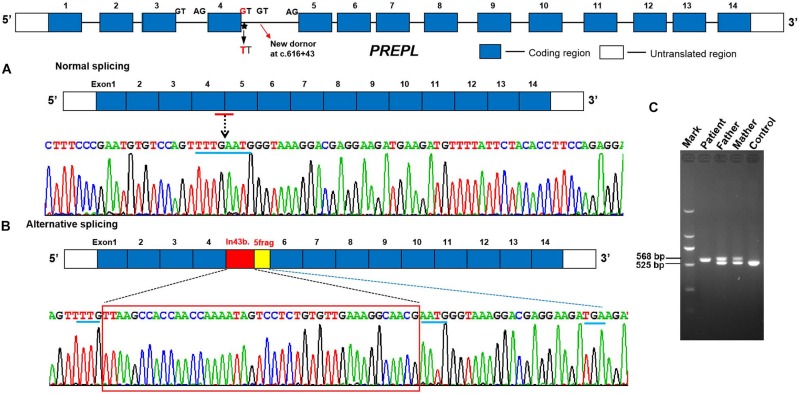
Aberrant mRNA transcripts were detected in peripheral blood cell from the proband and her parents. **(A)** Normal splicing between adjacent exons (control). **(B,C)** RT-PCR amplification and cDNA sequence chromatograms by Sanger sequencing of PREPL showing a lengthened 43 bp mRNA sequence of the 4 intronic sequence r.[616 + 1_616 + 43ins] and a frameshift which generate a PTC in exon 5 [p.(Glu206Glyfs*22)] in the proband and her parents compared with the unaffected control.

In order to analyze the effect of the splice site variant on mRNA processing, we performed cDNA analysis of total RNA from peripheral blood from patient. The amplification of exon 3 to 5 resulted in an additional larger fragment in the patient’s parents and the patient suggesting that the c.616 + 1G > T intronic variant activates a cryptic acceptor splice site within intron 4, and the insertion of intronic nucleotides in the PREPL mRNA (Figure 2C). cDNA sequence analysis further confirmed that the c.616 + 1G > T variant causes a shift from canonical splicing to cryptic splicing that leads to the inclusion of 43 nucleotides in the sequence of intron 4 r.[616 + 1_616 + 43ins]; and a frameshift s a premature termination codon in exon 5 [p.(Glu206Glyfs^∗^22) (Figures 2A,B)]. Collectively, this data provided crucial pathogenic evidence for the variant. According to ACMG standards, and guidelines for the interpretation of sequence variants, the novel mutation we describe here is pathogenic.

## Discussion

Hypotonia-cystinuria syndrome has been described as a disorder with cystinuria and congenital myasthenic result from the recessive deletions in *SLC3A1* and *PREPL* (Jaeken et al., 2006; Régal et al., 2014; Legati et al., 2016; Tucker et al., 2019). To date, only 10 patients with isolated PREPL deficiency (Jaeken et al., 2006; Régal et al., 2014, 2018; Laugwitz et al., 2018; Silva et al., 2018^6^) and 21 HCS families (contiguous deletion, including the *SLC3A1* and *PREPL* genes) have been reported (Clara and Lowenthal, 1966; Polgár, 2002; Font-Llitjós et al., 2005; Szeltner et al., 2005; García-Horsman et al., 2007; Martens et al., 2007; Boonen et al., 2011; Eggermann et al., 2012; Régal et al., 2012, 2014; Bartholdi et al., 2014; Wortmann et al., 2015). The most frequent characteristic features of subjects with PREPL deficiency are severe neonatal hypotonia, growth impairment and cognitive problems. In this study, we found a novel homozygous splicing mutation, c.616 + 1G > T, in the *PREPL* gene from a Chinese family using WES technology.

Further analysis by RT-PCR showed that the novel homozygous splicing mutation c.616 + 1G > T in the *PREPL* gene resulted in the insertion of 43 nucleotides in intron 4 and truncation of the N-terminal half of and C-terminal all of PREPL protein, thus leading to a loss of function. The analysis of several public databases showed that this homozygous splicing mutation was very rare. Therefore, this mutation of the *PREPL* gene is pathogenic and leads to a PREPL deficiency phenotype. As previously, subjects with PREPL deficiency often present with growth deficiency. Growth hormone therapy has a positive effect on the cohort of cases that exhibit growth hormone deficiency (Jaeken et al., 2006; Régal et al., 2014, 2018). In our study, the patient suffered congenital myasthenic syndrome, a failure to thrive, severe growth deficiency, and short stature. These symptoms were partly related to the delayed diagnosis and the consequential lack of growth hormone therapy. Patients with PREPL deficiency often develop obesity due to hyperphagia in late childhood; however, our patient was 14 with a normal BMI at our initial diagnosis. In a previous study, Silva et al. (2018) observed moderate ID patients, and showed that biallelic *PREPL* mutations alone (without involvement of other genes) can cause ID. In the present study, our patient also present with ID, and a homozygous point mutation of c.616 + 1G > T.

Our patient showed the common phenotypes associated with PREPL deficiency, including severe neonatal hypotonia, delayed independent walking, mental retardation and neonatal feeding problems. However, we also observed additional phenotypic features. Previous studies of patients with HCS and isolated PREPL deficiency patients have reported the involvement of the brain and skeletal muscle tissues; these factors are consistent with the mental retardation and hypotonia observed in such patients. However, such literature does not refer to the absence of ovaries and hypoplasia of the uterus. Although hypergonadotropic hypogonadism has been observed in some patients with isolated PREPL deficiency (Richards et al., 2015; Régal et al., 2018), this study is the first to report the absence of the ovaries and hypoplasia of the uterus. We also observed other symptoms in our patient including microcephaly and a short neck. PREPL is considered to be the member of prolyl oligopeptidase subfamily of serine peptidases. Prolyl peptidases potentially have the capacity to participate in a variety of cellular regulatory processes, as their substrates are involved in regulating different signaling pathways (Polgár, 2002; García-Horsman et al., 2007). Previous studies have reported that PREPL is expressed widely across many tissue types (Jaeken et al., 2006; Boonen et al., 2011). The established function of PREPL is to redistribute clathrin-associated adaptor protein complex 1(AP-1) from the cell membrane into the cytoplasm; AP-1 mediates the transport of vesicular acetylcholine transporter (Lone et al., 2014). PREPL deficiency causes a reduction in the filling of ACh synaptic vesicles (Régal et al., 2014). This suggests that PREPL might act on many types of cells and participate in a series of biological processes, including cell division, apoptosis, cell differentiation, and functional activities involving oestrogens and uterus. The mutation in the of PREPL gene may affect the function of PREPL protein in cellular biological processes to varying degrees, and therefore cause distinct outcomes in different individuals and tissues.

## Conclusion

In conclusion, we identified a point homozygous mutation (c.616 + 1G > T) in the PREPL gene in a Chinese girl with typical PREPL deficiency. The girl exhibited the normal symptoms of PREPL deficiency, including severe neonatal hypotonia, growth impairment and cognitive problems, however, she also suffered from hypergonadotropic hypogonadism, along with uterine and ovarian abnormalities. Collectively, these symptoms can be used as the future basis for diagnosis. In future, detailed molecular and clinical features are likely to be useful for extending the evidence for genetic and phenotypic heterogeneity and exploring the phenotype-genotype correlations in isolated PREPL deficiency.

## Data Availability Statement

We have uploaded the DNA sequencing data to SRA public repository in NCBI SRA accession: PRJNA606978 (https://www.ncbi.nlm.nih.gov/sra/PRJNA606978).

## Ethics Statement

All procedures in this study were approved by the Institutional Review Boards and Ethics Committees of Guangxi Maternal and Child Health Hospital. Detailed written informed consent was obtained from all participants.

## Author Contributions

QY and XF designed the study and drafted the manuscript. RH, QY, JQ, SaY, FS, and ML extracted, analyzed, interpreted the data, and collected the clinical data. QZ, XF, SeY, and ML performed the targeted sequencing, analyzed, and interpreted the data. JQ and RH participated in the study coordination and revised the manuscript. All authors read and approved the final version of the manuscript.

## Conflict of Interest

The authors declare that the research was conducted in the absence of any commercial or financial relationships that could be construed as a potential conflict of interest.

## Footnotes

1 http://www.hgmd.cf.ac.uk/ac/all.php

2 http://sift.bii.a-star.edu.sg/www/SIFT_seq_submit2.html

3 http://genetics.bwh.harvard.edu/pph2/

4 http://www.mutationtaster.org/

5 http://www.umd.be/HSF/

6 www.hgmd.cf.ac.uk/ac/gene
